# Advanced Infill Designs for 3D Printed Shape-Memory Components

**DOI:** 10.3390/mi12101225

**Published:** 2021-10-08

**Authors:** Daniel Koske, Andrea Ehrmann

**Affiliations:** Faculty of Engineering and Mathematics, Bielefeld University of Applied Sciences, 33619 Bielefeld, Germany; daniel.koske@fh-bielefeld.de

**Keywords:** 3D printing, poly(lactic acid) (PLA), fused deposition modeling (FDM), shape-memory polymer (SMP), 4D printing, infill patterns

## Abstract

Poly(lactic acid) (PLA) is one of the most often used polymers in 3D printing based on the fused deposition modeling (FDM) method. On the other hand, PLA is also a shape memory polymer (SMP) with a relatively low glass transition temperature of ~60 °C, depending on the exact material composition. This enables, on the one hand, so-called 4D printing, i.e., printing flat objects which are deformed afterwards by heating them above the glass transition temperature, shaping them and cooling them down in the desired shape. On the other hand, objects from PLA which have been erroneously deformed, e.g., bumpers during an accident, can recover their original shape to a certain amount, depending on the applied temperature, the number of deformation cycles, and especially on the number of broken connections inside the object. Here, we report on an extension of a previous study, investigating optimized infill designs which avoid breaking in 3-point bending tests and thus allow for multiple repeated destruction and recovery cycles with only a small loss in maximum force at a certain deflection.

## 1. Introduction

Nowadays, 3D printing is used for many applications, from rapid prototyping to rapid tooling to rapid manufacturing [1]. Printing polymers is often performed by the fused deposition modeling (FDM) technology which enables creating 3D shapes from a broad variety of polymers [2,3]. One of the most often used polymers in FDM printing is poly(lactic acid), a biodegradable natural polymer without cell-toxic properties and thus also suitable for biotechnological and biomedical applications [4,5].

While 3D printing in general allows for creating nearly all shapes, in many cases this freedom of design is limited by technical problems, such as the requirements to have a suitable contact surface with the printing bed, to avoid too strong overhangs, etc. This is why in many cases it would be supportive to print a certain shape flat on the printing bed and afterwards deform it to reach the final shape. PLA indeed shows such shape-memory functionality [6,7,8]. It can thus be used to fit orthoses to an individual finger [9], but also for diverse biomedical applications, such as for minimally invasive surgeries [10,11,12,13]. 4D printing PLA is also used for design purposes and other applications [14,15,16,17].

In case of 4D printing, the required final shape is reached by heating the as-printed object to a temperature above the glass transition temperature, i.e., typically in the range of 60 °C to 100 °C in case of PLA [6,10,11], deforming the object at high temperature and fixing the desired shape during cooling it down back to room temperature. Another application of shape memory polymers, however, is related to accidental deformation, e.g., of bumpers, safety equipment for sports, etc. In this case, the deformation occurs at low temperatures where the mechanical impact may not only lead to reversible deformation, but also to broken bonds between 3D printed lines or within them.

In a previous study, we thus investigated diverse infill patterns, comparing some of the patterns available in slicing programs for FDM printers with self-designed infill patterns, mostly based on the idea of leaf spring structures [18]. Here, we show the results of a subsequent study, leading to more sophisticated structures which retain their shapes to a large amount even after 10 bending and recovery cycles.

## 2. Materials and Methods

The samples used in this study were printed using a MEGA-S FDM 3D printer (ANYCUBIC; Shenzhen Anycubic Technology Co., Ltd., Shenzhen, China). A nozzle diameter of 0.4 mm was used to set a layer thickness of 0.2 mm for the first layer and 0.12 mm for the other layers. The printing temperature was set to 200 °C and the heating bed temperature was set to 60 °C constantly.

The test specimens made of PLA (GIANTARM, Shenzhen, China; PLA filament 1.75 mm, silver; without additives to increase crystallinity) were constructed with the dimensions of 120 mm × 15 mm × 6 mm following polymer test specifications (ISO 20753:2018) and printed on the long side (on the rear of the CAD models shown in Table 1). 

For the three-point bending test, the specimens were prepared with two completely filled plates on top and bottom, each 1 mm thick, to support the internal structure as a base support. No wall structures were implemented in order to more accurately test the behavior of the infill specimens. This basic structure was retained for all test specimens, only the infill structure was changed.

All specimens were designed and constructed in-house and always printed under the same circumstances, with a sample fill density of 100%, a print speed of 50%, and a high edge print positioning with a supporting brim with 6 line counts. To illustrate the material content, the mass of the test specimens was determined using a Xavax Jewel digital precision balance (Hama GmbH & Co KG, Monheim, Germany) and used as an indication and comparison value (Table 1).

In the first phase, certain basic patterns have been developed as filling structures for the specimen. The structures of the test specimen series “A” were created following an Octet grid matrix pattern [19]. A sample A-k with smaller repeat unit and a sample A-G with larger repeated unit were designed and printed, optimizing the structures presented in [18].

Test specimen “B” is an extension from the self-developed structure “LP100” from [18]. Here, waves and textile structures were combined for a new structural idea.

The infill pattern for test specimens “C” includes the structural fabric from dynamic lightweight design [20]. Auxetic structural behavior [21] is to be tested for shape memory effects in the test specimens C-k and compared with the other designs.

In the second phase, the filling patterns of the first phase have been changed or mixed. The AC-G test specimen was created as a mixture of A-G and C-k. The AC-H test specimen was designed from the A-G pattern, with the inclusion of reinforced structures due to increased fill pattern content. The specimen D-G is another new approach constructed from the test specimen series “A” and “C”.

Each sample was tested in triplicates regarding maximum deflection and with different amounts of specimens, as given in the Results section, for bending and recovery tests.

Bending and recovery tests were performed by a universal testing machine (Kern & Sohn, Balingen-Frommern, Germany). While each set of samples was first tested until break (or until the maximum deflection possible in the test setup) to find the point of maximum force, recovery tests were performed until the maximum force was reached, followed by recovery in a water bath of (60 ± 1) °C for 1 min, before it was cooled down in another water bath at room temperature for 2 min. This temperature is above the glass transition temperature of amorphous PLA used for 3D printing [22,23].

The main differences to the previous study [18] are as follows:

- The inner walls are thinner, enabled by printing on a long thin plane (front or back in the sliced models in Table 1), thus reducing the specimen’s mass.
- Channels along the whole specimen are not only produced along the shorter in-plane direction (from front to back in Table 1), but also along the longer in-plane direction (from left to right in Table 1).
- Special lightweight designs are used (samples A and C), again reducing the specimen’s mass.
- All samples are more lightweight than the best specimen of the previous study (sample LP100 in [18] had a mass of 9.8 g).

## 3. Results and Discussion

**Figure 1:**
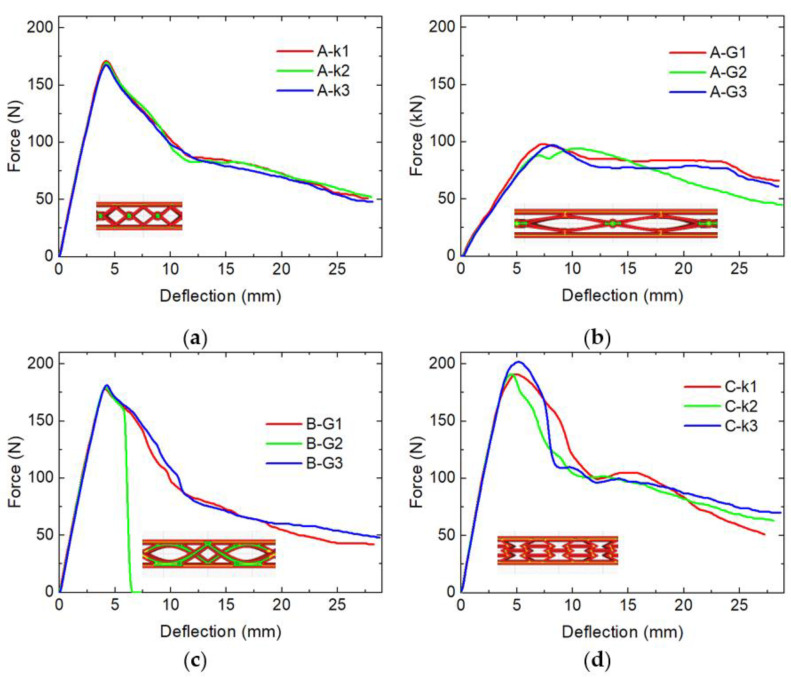
Maximum bending tests of the first set of samples: (**a**) specimens A-k; (**b**) specimens A-G; (**c**) specimens B-G.; (**d**) specimens C-k.

First, Figure 1 depicts the results of the maximum bending tests. Generally, the three nominally identical samples behaved quite similarly in most cases, with one specimen of sample B-G (Figure 1c) breaking during the test, while the others remained intact.

For samples A-k, B-G, and C-k, a maximum bending force is visible near 5 mm deflection, followed by a decrease of the force necessary for further bending up to a deflection of ~10 mm, and a subsequent further decrease with smaller slope until the maximum possible deflection was reached. In these three samples, the maximum forces reached values around 170–200 N.

Sample A-G behaves slightly differently. Here, the maximum force is lower, is reached later, and the slope of the elastic part of the curve (around 0–6 mm) is lower. As the infill percentage varies from one position to the other in these samples, calculation of the elastic modulus from these forces is not unique and thus not used for comparison here.

More interestingly, in sample A-G the force does not strongly decrease after the maximum value but stays on a similar level in two of the three samples under investigation and slowly decreases in the third one. It must be mentioned that in this sample, the pattern has the largest lateral dimensions of the unit cell, i.e., small deviations of the positioning in the test stand can have a large influence on the result.

This relatively constant level of force, independent from the deflection, can be assumed to be advantageous for the planned application in recoverable bumpers etc., where the deformation depth will vary, and a material structure able to absorb energy constantly along a broad range of deformation is necessary.

**Figure 2:**
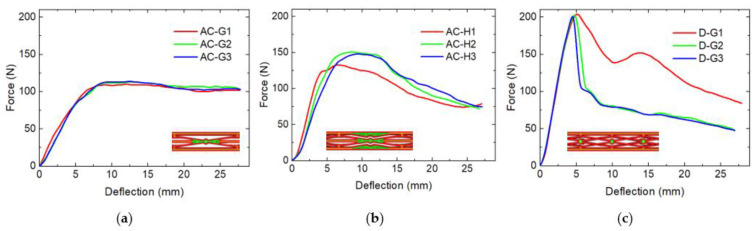
Maximum bending tests of the improved set of samples: (**a**) specimens AC-G; (**b**) specimens AC-H; (**c**) specimens D-G.

This is why more structures similar to the infill pattern A-G were prepared, which are presented in Figure 2.

Table 2 depicts the maximum forces as well as absorbed energies for the maximum deflection of ~27.5 mm used in these tests [24]. Due to the small dimensions of the tested specimens, the absorbed energies are not large, but allow for comparing the different samples, showing that the absorbed energies are similar for most samples, with samples A-G absorbing the smallest energy and the last four samples (C-k, AC-G, AC-H, and D-G) absorbing significantly more energy than the others. Among these four samples, AC-G has the smallest maximum force, i.e., would cause the smallest impact on the opposite object in a crash and seems thus advantageous for the possible use as a bumper.

While the last of these improved samples (Figure 2c) again shows a sharp peak around 5 mm, similar to sample A-k, sample AC-H shows a much broader peak at larger deflection (Figure 2b), and sample AC-G shows a nearly constant force from ~9 mm up to the maximum available deflection. For the idea of preparing bumpers, sample AC-G would thus be ideal.

**Figure 3:**
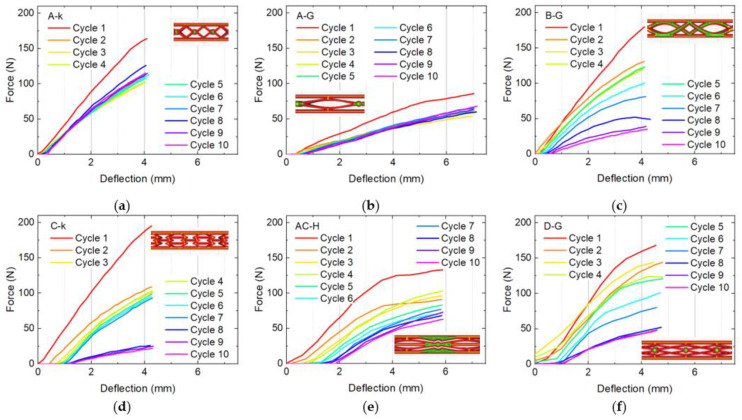
Bending and recovery tests of samples with different infill patterns: (**a**) A-k; (**b**) A-G; (**c**) B-G; (**d**) C-k; (**e**) AC-H; (**f**) D-G.

Next, Figure 3 shows the recovery tests up to the deflection where the force became maximum in the previous tests. As described above, well-applicable shape memory polymer samples should be able to recover their original shape, which is visible from the position where the subsequent cycles start; the stronger the residual deformation is, the larger is the deflection where the force is for the first time larger than zero. On the other hand, good recovery properties mean that subsequent cycles show similar forces at maximum deflection.

According to these rules, a clear pre-selection among the here tested samples can be made. The specimens C-k and AC-H show a relatively strong residual deformation, i.e., not a good recovery. Besides, in sample C-k there are severe “jumps”, resulting from large broken bonds in this sample, making this structure not well usable. 

The samples B-G and D-G, on the other hand, show strongly reduced maximum forces, in both cases losing more than 2/3 of the original maximum force after 10 bending and recovery cycles. This makes these two samples not well suited for applications in bumpers, etc. either. 

The samples A-k and A-G, on the other hand, do not show both these problems. While in both cases there is a clear difference between the first and the second cycle, all subsequent cycles show relatively similar values, in case of sample A-k even with a small increase of the maximum force from the fifth cycle which may be due to work hardening [25]. However, sample A-k showed only a narrow peak, indicating that larger deformation will not be recovered in a similar way.

**Figure 4:**
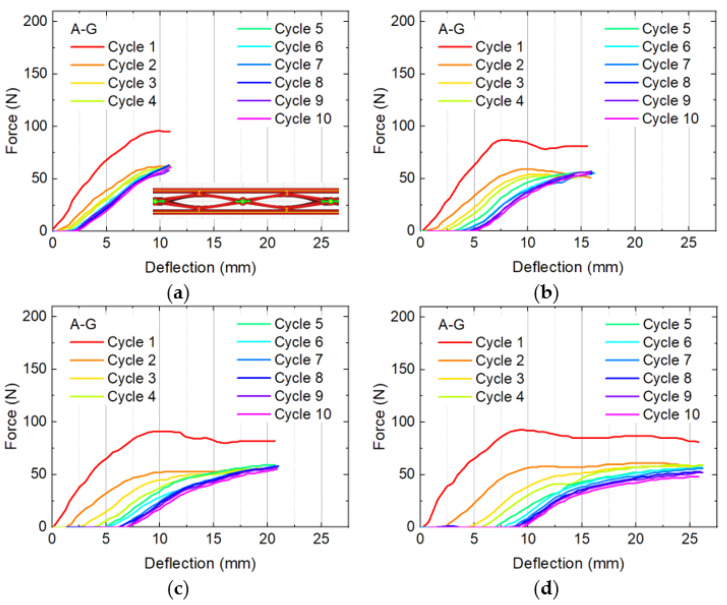
Bending and recovery tests of four A-G specimens with different maximum deflections: (**a**) 11 mm; (**b**) 16 mm; (**c**) 21 mm; (**d**) 26 mm.

Thus, Figure 4 shows bending and recovery cycles of sample A-G, which was found to have only a slight decrease of the force for larger deflections, now comparing maximum deflections of approximately 11 mm, 16 mm, 21 mm, and 26 mm, i.e., testing in steps of ~ 5 mm between the maximum force (Figure 3b) and the maximum possible bending.

In comparison with the first recovery test, using a maximum deflection of 7 mm (Abb. 3b), here the difference between the maximum forces of the first and the residual cycles is larger. Besides, the residual deformation grows with the maximum elongation, as it could be expected. On the other hand, in all cases the last cycles show a relatively constant behavior in terms of maximum force and residual deformation. Again, a slight work hardening is visible where lines of subsequent tests cycles are crossing. No breaking of the samples occurred, making it in general suitable for safety applications etc.

**Figure 5:**
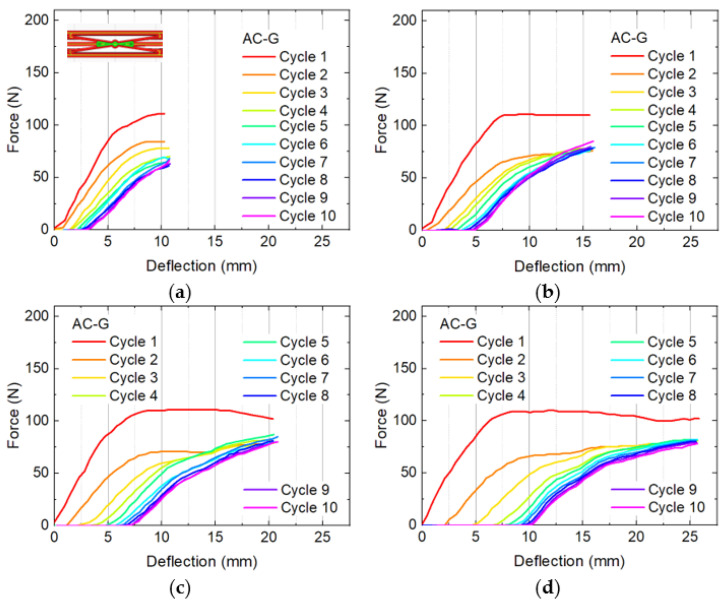
Bending and recovery tests of four AC-G specimens with different maximum deflections: (**a**) 11 mm; (**b**) 16 mm; (**c**) 21 mm; (**d**) 26 mm.

Very similar findings can be recognized in Figure 5 where the same tests are depicted for sample AC-G. Comparing the recovery tests of both samples A-G and AC-G, they show comparable maximum forces, with higher values for sample AC-G, nearly identical residual deformation, and slightly smaller differences between the forces during the first cycle and the subsequent ones for sample AC-G. As Table 1 shows, both samples share a similar idea of construction. This indicates that this sort of construction, further optimized, is advantages in comparison with the other structures tested here.

Finally, in comparison with the previous structures [18], it must be mentioned that the latter partly showed larger maximum forces, similar residual deformation, but a stronger reduction of the maximum force with subsequent cycles, indicating that the recent structures are a better base for applications in bumpers and other reusable safety equipment.

**Figure 6:**
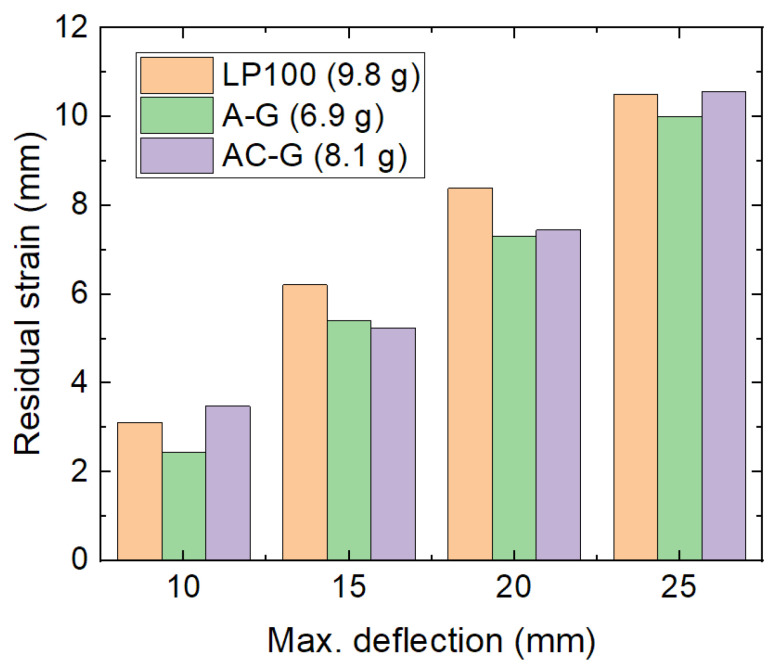
Comparison between the best lightweight samples of the recent study with the best (heavier) sample of the previous study [18] in terms of the residual strain.

The main aim of this study was to find more lightweight structures with identical or better recovery properties as the best sample of the previous study [18]. Figure 6 thus compares the residual strain of the samples A-G and AC-G (Figure 4 and Figure 5) with the results of the sample LP100 from the previous study (Figure 7b in [18]). It is clearly visible that the most lightweight sample, A-G, does not show larger residual strains than the best sample of the previous study and for most maximum deflections similar results as the heavier sample AC-G. The structure A-G is thus best suited for a lightweight recoverable object.

## 4. Conclusions

Different self-designed infill structures were used to prepare 3-point bending test samples from PLA by 3D printing via the FDM technique. Two of the samples designed as typical lightweight structures showed relatively constant forces for a broad range of deflection, making them suitable for safety applications. Bending and recovery tests revealed good recovery properties of these samples after heating the specimens to 60 °C, with one of the lightweight structures designed for the recent studies showing even lower residual strain despite significantly reduced mass. Next, the recovery process itself must be optimized in terms of temperature and duration, besides the choice of the ideal PLA material, combined with a final optimization of the specimen geometry, before tests with fast deformations will be performed, as they will typically occur during accidents.

## Figures and Tables

**Table 1:** Samples used in this study. Red parts denote the printed shell, the green parts show the printed inner wall (all samples were printed without support structures).

Sample Name (Sample Mass)	Sliced Model	Printed Specimen
A-k (6.1 g)	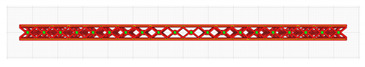	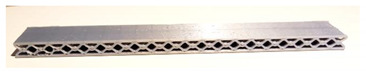
A-G (6.9 g)	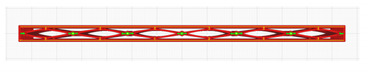	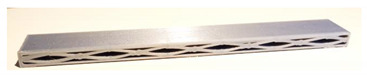
B-G (6.6 g)	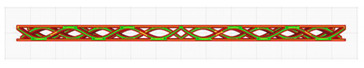	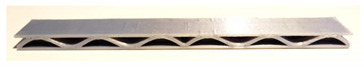
C-k (9.0 g)	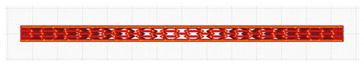	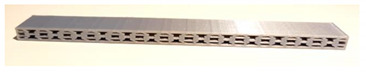
AC-G (8.1 g)	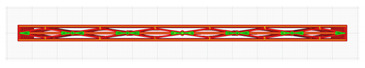	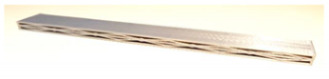
AC-H (9.4 g)	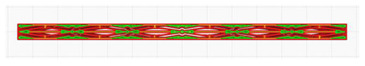	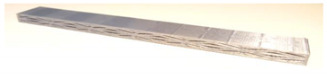
D-G (9.2 g)	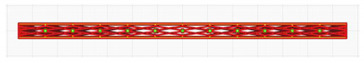	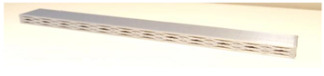

**Table 2:** Maximum forces and absorbed energies derived from Figure 1 and Figure 2.

Sample	Max. Force (N)	Absorbed Energy (J)
A-k	169.7 ± 1.5	2.47 ± 0.04
A-G	96.3 ± 2.1	1.99 ± 0.12
B-G	180.0 ± 1.7	2.42 ± 0.09 ^1^
C-k	195 ± 6	2.91 ± 0.10
AC-G	112.3 ± 2.1	2.622 ± 0.010
AC-H	144 ± 10	2.73 ± 0.12
D-G	206.0 ± 2.0	2.6 ± 0.8

^1^ Calculated without B-G2 which broke during the test.

## Data Availability

The data presented in this study are fully available in this article.
